# Community-Based Brain Health Promotion During the COVID-19 Pandemic: Efforts of AARP in the USA

**DOI:** 10.1093/geroni/igab046.1875

**Published:** 2021-12-17

**Authors:** Sarah Lock, Lindsay Chura, Rachel Lazarus

**Affiliations:** 1 AARP, Washington, District of Columbia, United States; 2 Staying Sharp, AARP, Washington, District of Columbia, United States

## Abstract

The pandemic has created new barriers for the delivery of healthcare resources and information, as well as in-person delivery of health care, caregiving, and social engagement. AARP created trainings designed for volunteer-led community-based brain health promotion. Due to the COVID, we have had to convert them to virtual presentations, distributed through technology. Staying Sharp is AARP’s online platform that educates users about integrated, holistic, lifestyle-based approaches for maintaining brain health as we age. This platform was created for convenience and scalability – we currently have over 800,000 users. During a period of necessary isolation, this platform has performed as an ideal way to get helpful information about maintaining brain health to our consumers, who are now stuck at home without access to in-person (e.g., community-based) alternatives. We will discuss lessons learned from these two different approaches along with preliminary data on behavior change based on these engagements.

